# Browning Epicardial Adipose Tissue: Friend or Foe?

**DOI:** 10.3390/cells11060991

**Published:** 2022-03-14

**Authors:** Elisa Doukbi, Astrid Soghomonian, Coralie Sengenès, Shaista Ahmed, Patricia Ancel, Anne Dutour, Bénédicte Gaborit

**Affiliations:** 1 INSERM, INRAE, C2VN, Aix-Marseille University, F-13005 Marseille, France; elisa.doukbi@univ-amu.fr (E.D.); astrid.soghomonian@gmail.com (A.S.); shaistaahmed.sh@gmail.com (S.A.); patricia.ancel@univ-amu.fr (P.A.); anne.dutour@ap-hm.fr (A.D.); 2 Department of Endocrinology, Metabolic Diseases and Nutrition, Pôle ENDO, APHM, F-13005 Marseille, France; 3 Stromalab, CNRS ERL5311, EFS, INP-ENVT, INSERM U1031, University of Toulouse, F-31100 Toulouse, France; coralie.sengenes@inserm.fr; 4 Institut National de la Santé et de la Recherche Médicale, University Paul Sabatier, F-31100 Toulouse, France

**Keywords:** epicardial adipose tissue, ectopic fat, immune cells, beiging, browning, adipose tissue, heart, coronary artery disease, innate lymphoid cells

## Abstract

The epicardial adipose tissue (EAT) is the visceral fat depot of the heart which is highly plastic and in direct contact with myocardium and coronary arteries. Because of its singular proximity with the myocardium, the adipokines and pro-inflammatory molecules secreted by this tissue may directly affect the metabolism of the heart and coronary arteries. Its accumulation, measured by recent new non-invasive imaging modalities, has been prospectively associated with the onset and progression of coronary artery disease (CAD) and atrial fibrillation in humans. Recent studies have shown that EAT exhibits beige fat-like features, and express uncoupling protein 1 (UCP-1) at both mRNA and protein levels. However, this thermogenic potential could be lost with age, obesity and CAD. Here we provide an overview of the physiological and pathophysiological relevance of EAT and further discuss whether its thermogenic properties may serve as a target for obesity therapeutic management with a specific focus on the role of immune cells in this beiging phenomenon.

## 1. Introduction

Epicardial adipose tissue (EAT) is a visceral adipose tissue located between the myocardium and the inner layer of the pericardium. EAT is characterized by its cardioprotective functions in physiological conditions and its increased expression of thermogenic genes giving its adipocytes a beige/brite phenotype. This metabolically active tissue has endocrine properties, and a special location that makes it able to modulate the structure and function of the myocardium. In many clinical studies, EAT has been associated with the appearance of cardiovascular diseases such as atrial fibrillation (AF) and coronary artery disease (CAD), but also with their severity [1]. In recent years, non-invasive imaging studies revealed that addition of artificial intelligence (AI) deep learning approaches EAT quantification to current risk assessment tools resulted in a significant net reclassification improvement for major adverse cardiovascular events (MACE). Transcriptional studies revealed that EAT exhibits high expression of the beige adipocyte-specific marker CD137, and also thermogenic genes such as UCP-1, PRDM16, PGC-1α, PPARγ and BAT-specific genes such as actin alpha 1 (ACTA1), PPARγ co-activator 1 alpha (PPARGC1A), troponin C type 1, and troponin I type 1 compared to the subcutaneous adipose tissue (SAT) [2,3]. On the other hand, EAT beiging or browning phenomenon could have a benefit effect on cardiovascular diseases. Beiging process is the appearance of beige cells with thermogenic function in the white adipose tissue (WAT), that contributes to energy expenditure through nonshivering thermogenesis [4,5,6]. Many cellular and molecular actors have been shown to participate in the browning of WAT such as mastocytes, eosinophils, alternatively activated macrophages, innate lymphoid cells; IL-13, IL-5, IL-4, amphiregulins, methionine/enkephalin, atrial natriuretic peptides (ANP), and could represent future anti-obese therapeutic targets. However, far less is known about the loss of EAT browning with age, obesity, or CAD.

In this review, we will focus on the beiging or browning of EAT in cardiovascular and metabolic diseases and its link with immune cells.

## 2. The Epicardial Adipose Tissue

### 2.1. Physiological Features

The EAT is an ectopic fat depot located between the myocardium and the visceral pericardium. The close proximity between the adipose and the cardiac tissue allows functional and anatomical relationships. Both share the same microcirculation, with no fascia separating the two layers, allowing cellular exchanges [7]. Moreover, the EAT represents 20% of the heart weight under physiological conditions [8,9]. On the other hand, in terms of total fat mass it represents only 1%, which is far from the mass of the abdominal visceral adipose tissue (VAT) [9]. Among the several functions of this tissue, there is a mechanical function. Indeed, it can protect coronary arteries against torsion induced by the arterial pulse wave and cardiac contraction [9]. Another putative function related to the EAT is its local energy storage for the heart, but also its protective role against elevated levels of free fatty acids (FFAs) in the microcirculation [10]. This function is very important since the myocardium metabolizes FFAs from the coronary arterial blood, and their oxidation is responsible for about 50–70% of the energy production of the heart, so EAT can be seen as a buffer to protect the heart against lipotoxicity or lack of energetic substrate [7]. Besides, the rate of fatty acids (FA) release by EAT is approximately twice that of other fat depots [11]. It is also possible to distinguish EAT from other visceral fat depots by its higher capacity to uptake FFAs and its lower rate of glucose utilization, suggesting a high plasticity in lipid metabolism activity for this ectopic fat depot [12]. 

Epicardial adipocytes are smaller than subcutaneous and visceral adipocytes [13] and one of the particularities of these adipocytes is their beige phenotype [3]. It has been hypothesized to function like brown adipose tissue and generate heat in response to cold temperatures and activate the autonomic nervous system [14,15]. This leads to another putative function of the EAT which is the protection of the heart against hypothermia. We will discover next in this review that the beige character of EAT provides many opportunities to better understand its physiological role and to discover new molecular targets to prevent CVD.

### 2.2. EAT as an Endocrine Organ

More than a fat depot, EAT is increasingly recognized as an endocrine organ being a source of many bioactive molecules that can modulate the myocardium’s and coronary arteries’ homeostasis [16,17,18,19]. Two main hypotheses exist to explain how EAT-derived secreted molecules can interact with coronary arteries [20,21]. First, the paracrine signaling which assumes that EAT-derived adipokines diffuse directly through the layers (adventitia, media and intima) of the vascular wall via the interstitial fluid to interact with smooth muscle [17,22,23]. Then, the vasocrine signaling hypothesis, which implies that adipokines and FFAs directly enter into the vasa vasorum and are transported downstream into the arterial wall [20,24]. But more recently, a new communication mode has been evidenced implicating extracellular vesicles (EVs) containing various cytokines and microRNAs [25]. In this article, Shaihov-Teper et al. demonstrated that EAT is able to address EVs carrying proinflammatory, profibrotic and proarrhythmic molecules to the atria. 

**Figure 1:**
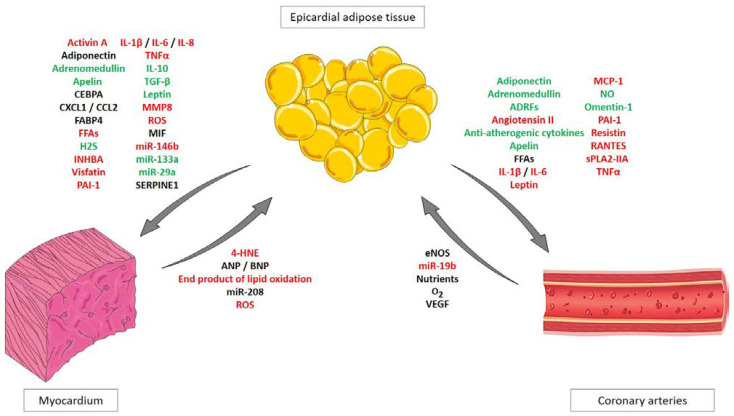
The crosstalk between epicardial adipose tissue and the cardiovascular system. A local crosstalk takes place between EAT (epicardial adipocytes and other EAT composing cells) and the cardiovascular system (e.g., myocardium and coronary arteries). Physiological and pathophysiological signals such as cytokines, adipokines or fibrokines can be released from EAT to cardiomyocytes (and from myocardium to EAT) or coronary artery endothelial cells (grey arrows). Theses cytokines can have protective and/or beneficial effects (green) or harmful effects (red). Other molecules’ effects are still not well established or are controversial (black).

Among these bioactive molecules interleukins (IL) (IL-1β, IL-6, IL-8, IL10), adiponectin, plasminogen activator inhibitor 1 (PAI-1), adrenomedullin, phospholipase A2, tumor necrosis factor α (TNF-α), monocyte chemoattractive protein 1 (MCP-1), omentin, leptin, visfatin, resistin have been identified (Figure 1) [26,27,28,29]. Although some of these molecules have physiologically positive effects, such as adiponectin, or omentin, the imbalance between protective and deleterious adipokines secreted by EAT may participate in the proinflammatory phenotype associated with endothelial dysfunction and atherogenesis [30]. Indeed, under physiological conditions some adipokines secreted by the epicardial fat such as adiponectin, adrenomedullin and omentin are supposed to protect the vasculature from oxidative stress or regulate the arterial vascular tone and improve endothelial function [9]. Adrenomedullin and its receptors expressed in EAT are regulated by coronary status and this hormone can play a cardioprotective role by inhibiting oxidative stress [27]. By contrast, harmful effects of pro-inflammatory cytokines expressed by EAT such as IL-1β, IL-6, TNFα, PAI-1, MCP-1 have also been reported [30]. In 2008, Cheng et al. showed that tissue levels of leptin, visfatin, IL-6 and TNFα were higher in EAT from CAD patients compared to non-CAD patients [31]. Increased levels of TNF or its soluble receptors have been implicated in ischemia-reperfusion injury, myocarditis, cardiac allograft and also in the progression of congestive heart failure [22,32,33]. Adipo-fibrokines such as activin-A or matrix metalloproteinases (MMPs) also participate in extracellular matrix remodeling and could be involved in fibrogenesis [24]. On the other hand, not only can the EAT influence the heart and coronary arteries, EAT is also able to receive biological signals from the heart and thus in return will be able to modify its secretome, indicating a crosstalk between EAT and the cardiovascular system [34]. In particular, researchers noticed in EAT that the expression of the gene encoding for adiponectin *ADIPOQ* was positively associated with myocardial oxidative stress [34,35] probably through an adaptive mechanism. Usually, an increased expression of *ADIPOQ* is correlated to a reduced myocardial nicotinamide adenine dinucleotide phosphate oxidase-derived O_2_ (−) production. But in the study of Antonopoulos and colleagues, the crosstalk between myocardium and EAT translates into the induction of O_2_ (−) in H9C2 cardiomyocytes. This results in the production of transferable factors that up-regulate adiponectin expression in EAT via peroxisome proliferator-activated receptor γ (PPAR-γ) [35]. This work showed that not only can EAT communicate with the myocardium, but the opposite also exists.

### 2.3. Immune Cellular Composition of EAT

Because of the singular proximity between the myocardium and the EAT, the adipokines and pro-inflammatory molecules secreted by the EAT may directly affect the metabolism of the heart and coronary arteries. Its particular secretome (epicardial adipokines) switches to a proinflammatory profile in obesity and CAD and can induce atherogenic changes in monocytes and endothelial cells [36,37,38,39,40]. Thus, inflammation could be linked to an unbalance in immune cells. In this part we will develop what is known about immune cells in EAT and how immune cells may also induce EAT extracellular matrix remodeling and could also have a role in EAT browning.

The epicardial stroma vascular fraction includes numerous nervous, nodal, and inflammatory or non-inflammatory immune cells in addition to stromal cells [7,41]. Among cells of the immune system present in the EAT, adaptive cells can be found, notably T and B lymphocytes, as well as innate cells such as macrophages, mast cells, and dendritic cells. Table 1 provides a unique comparison of the presence or absence of immune cell subtypes infiltration in the EAT and VAT compared to SAT in mice and humans in the context of obesity.

First, among phagocytic cells, macrophages are the most abundant immune cells found in the adipose tissue of mice and humans [42]. It is reported that macrophage can be present in adipose tissue in two different states i.e., pro-inflammatory classically activated known as M1; or anti-inflammatory alternatively known as M2 [43]. Aron-Wisnewsky et al. showed increased CD40 (M1) and reduced CD206 (M2) macrophages in VAT compared to SAT [44]. In EAT, studies have shown the existence of macrophage infiltration in the tissue, linking their polarization with the coronary status of patients [45,46,47]. Remarkably, Hirata et al. characterized the phenotype of macrophages and demonstrated that M1 macrophages are increased and M2 macrophages are decreased in EAT of CAD compared to non-CAD patients [45]. Recently, a study has also suggested that an elevated number of macrophages appeared to be associated with severe deterioration of heart function in CAD patients [23]. 

Other innate cells are present in EAT, among them, dendritic cells, (DCs) which can be subdivided into two categories: conventional DCs (cDCs) and plasmacytoid DCs (pDCs). cDCs initiate primary T-cell responses, thereby orchestrating adaptive immunity. In contrast, pDCs do not stimulate naive T cells, but can be converted into cDC-like cells upon activation and function as antigen presenting cells (APCs) [48]. DCs are significantly increased in obesity, and promote macrophage infiltration in adipose tissue and liver [49]. In EAT, Mráz et al., reported for the first time in 2019 the presence of both types of DCs in human EAT [50]. They also demonstrated that pDCs were significantly increased in EAT of type 2 diabetic (T2D) compared to non-T2D patients. 

Mast cells are commonly known for their role in several inflammatory and fibrotic diseases. Upon staining mast cells with tryptase and chymase, high levels of these cells were detected in obese omental adipose tissue [51]. Mazurek et al. by immunohistochemistry demonstrated the presence of mast cells in EAT [41].

Natural killer cells (NK) are well-known for killing virus-infected cells and controlling cancers [52]. In adipose tissue, it is suggested that NK cells recruitment, proliferation, and activation is stimulated by adipose tissue-resident macrophage derived secretory products [53]. These recruited NK cells within the adipose tissue might stimulate MCP-1 expression, which in turn causes macrophage infiltration [54]. In their 2009 study, Mráz et al. also reported a reduced number of NK cells in EAT of both CAD and non-CAD patients. In contrast, natural killer T cells (NKT cells) represent a subset of T lymphocytes along with NK cell surface markers [55]. A subset of NKT cells termed invariant NKT cells (iNKT) in adipose tissue maintains inflammation in quiescent state and regulates homeostasis of other anti-inflammatory immune cells, including M2 macrophages and Treg cells [56]. Their possible presence in EAT remains to be determined.

Other unconventional cells are interesting and should be given attention in the EAT: the first ones are type 2 innate lymphoid cells (ILC2s). ILC2s can be identified in adipose tissue [57], where they secrete IL-5 and IL-13 known to maintain insulin sensitivity in lean mice through the recruitment of eosinophils by IL5, and maintenance of M2 macrophages by IL-13 [58,59]. ILC2s may also control certain features of energy balance in mice, as IL-33-deficient mice (which have reduced functional ILC2s) develop spontaneous weight gain and fat mass on a low fat diet [57]. 

The other unconventional T cells that should be studied in EAT, but which have not been studied yet are gamma delta T cells (γδ T cells). In adipose tissue, they form between 5–15% of the total T cell compartment [60]. Kohlgruber et al. found that IL-17A-producing γδ T cells were the driving factor in promoting stromal-cell production of IL-33, which in turn promotes the maintenance of the adipose T-reg population. The authors further showed that mice lacking γδ T cells lacked the ability to regulate core body temperature after a cold challenge. Indeed, their data would suggest that γδ T cells/IL17A axis promotes thermogenesis.

Cells from the adaptive immune system are also present in adipose tissue; Nishimura et al. demonstrated an infiltration of CD8+ T Lymphocytes (LT) early in the development of obesity [61]. They also showed that transfer of CD8+ T cells into CD8-deficient obese mice induces M1 macrophage infiltration leading to adipose tissue inflammation. With regard to B lymphocytes (LB), they promote adipose tissue macrophage recruitment and TNF-α production in mice fed a high-fat diet (HFD). LB have also been linked with the accumulation and differentiation of IFN-γ-producing CD4+ and CD8+ T-cells in murine adipose tissue [62]. In EAT, Mráz et al. analyzed lymphocyte subtypes using cytometry and immunohistochemistry in SAT and EAT of patients with and without CAD [63]. They showed higher T cell content in EAT of subjects with CAD compared to non-CAD patients. Notably, they also demonstrated that LB even represent a higher percentage of total lymphocytes in EAT compared to SAT. However, a more comprehensive assessment of different T lymphocyte subpopulations in EAT, as well as their relation with CAD, is still lacking.

### 2.4. EAT in Heart Diseases

The special location of this adipose tissue allows it to communicate directly with the cardiomyocytes but also with the vascular wall of the coronary arteries. Recent evidence suggests2 that EAT plays an important part in the development of an unfavorable metabolic and cardiovascular risk profile [26,37,64,65]. Indeed, accumulation of fat around the heart is a well-established factor associated with the development of CVD, CAD, AF and heart failure.

#### 2.4.1. Coronary Artery Disease

Many studies have shown that EAT is significantly correlated with the extent and severity of CAD. In a large case-control study, the MESA (Multi-Ethnic Study of Atherosclerosis), increased EAT was associated with a higher risk of developing incident CAD in adult subjects with no history of CVD, which suggests a role of increased EAT volume to predict major coronary clinical events [66]. Some other case-control studies identified EAT volume as a strong predictor of myocardial ischemia [67] or flow-limit ischemia detected by fractional flow reserve [68]. In a recent prospective trial, Mahabadi et al. showed that EAT volume significantly predicted fatal and nonfatal coronary events independently of cardiovascular risk factors and CAC score (Coronary Artery Calcification score) [69]. EAT seems to be involved in early stages of atherogenesis. In a study using cold-pressor test, we previously showed, in healthy lean volunteers, a negative correlation between EAT amount and microvascular coronary vasodilation, an abnormality that can be detectable before the apparition of CAD, suggesting that EAT could be involved in endothelial dysfunction [70]. In asymptomatic subjects, EAT was associated with the presence and progression of coronary artery atherosclerosis especially in young subjects with low CAC score, suggesting that EAT may promote early atherosclerosis development [71,72]. 

All these findings support the hypothesis of the role of EAT in promoting the early stages of atherosclerotic plaque formation. The mechanisms by which EAT can cause atherosclerosis are complex and not completely understood. 

Epicardial fat might alter the coronary arteries through multiple pathways. Due to its secretion of bioactive inflammatory molecules, EAT is now recognized as involved in the formation of atherosclerotic plaques, and the onset of CAD [41,73]. Moreover, EAT in CAD patients showed increased expression of genes implicated in oxidative stress [74], and it is well established that the production of reactive oxygen species (ROS) impairs myocardial function and reduces the number of vital cardiomyocytes [75]. Numerous research teams have been working on EAT candidate molecules that could be involved in CAD. We previously demonstrated that the secretory type II phospholipase A2 (sPLA2-IIA), which has been shown to be an independent risk factor for CAD, showed an increased expression in EAT of CAD patients [26]. Other candidates such as catalase, carbonic anhydrase 1 (CAH1), phosphoglycerate mutase 1 (PGAM1), glutathione S-transferase P (GSTP1), and protein disulfide isomerase (PDIA1), that are related to oxidative stress pathways, were found to have proteomic differences in EAT compared to SAT from CAD patients [76]. All these proteins, except catalase and CAH1, were increased in EAT compared to SAT and ROS production was higher in EAT than SAT. But these findings are associative studies and the causality of EAT-derived ROS in CAD remains to be demonstrated. 

Furthermore, EAT could lead to endothelial dysfunction and vascular remodeling by secreting fatty acids and pro-inflammatory mediators and via inducing the adhesion of monocytes to endothelial cells and macrophage activation [38,45]. In vitro studies showed an increase of mast cells in the adventitia of coronary lesions [45]. Also, it should be noted that the presence of macrophages and mast cells in EAT could contribute to underlying vessel instability, which can lead to plaque rupture [77]. Very recent studies using machine learning approaches, artificial intelligence (AI), imaging and radiomic methods, have improved EAT functional characterization with the detection of perivascular adipose tissue inflammation and structural remodeling, that led to a striking improvement of cardiac risk prediction in high-risk individuals [78,79].

By all these mechanisms and as shown in clinical studies EAT volume is a strong independent predictor of CAD. However, a beneficial impact of the reduction of EAT quantity or immune cells infiltration in CAD remains to be proven. 

#### 2.4.2. Atrial Fibrillation

A growing body of evidence suggests that EAT can have a biological impact on cardiovascular tissues and could be implicated in the pathogenesis of AF [80,81,82,83]. Many studies have shown an association between EAT amount and the AF risk, severity and post ablation or electrical cardioversion recurrence. 

The increase of total EAT volume was found to be associated with the prevalence of AF. In a large study Thanassoulis et al. reported a significant correlation between EAT volume and AF risk, independently of other measures of adiposity, and this association was maintained after adjusting on other AF risk factors [84]. In the same way, Nakanishi et al. demonstrated that peri-atrial EAT volume predicted the development of new-onset AF in patients with CAD, independently of the presence of hypertension, diabetes or left atrial enlargement [82]. Moreover, numerous studies have shown that EAT, surrounding the atria in particular, was linked to AF recurrence after ablation therapy [82,85]. These studies suggest that EAT is an important determinant of the AF substrate and the presence of other cardiovascular risk factors does not weaken this link. 

The lack of fascia separating EAT from myocardium favors inflammatory infiltrates in the atrial wall, which could trigger arrhythmias [86,87]. EAT-secreted adipokines could contribute to structural remodeling of the atrial myocardium that promotes fibrosis. This remodeling could enhance the loss of cells connection, altering the propagation of the depolarizing wave and leading to conduction defects: formation of microcircuits and breakthrough of electrical impulses [88,89]. The amount of EAT could also exercise a mechanical effect on left ventricle (LV) and right ventricle (RV) filling and lead to an atria enlargement, which is one of the risk factors for AF [90]. 

Thus, EAT is an important determinant of AF which might predict the outcomes of rhythm control strategies and peri-atrial EAT volume assessment could contribute to the prevention and the management of AF, and especially in patients with CAD.

#### 2.4.3. Cardiac Morphology and Function

EAT is anatomically and clinically related to cardiac morphology and function. An increased amount of EAT has been associated with increased LV mass and abnormal right ventricle geometry or subclinical dysfunction [24]. In patients without CVD, LV mass is correlated with the EAT thickness measured by echocardiography [91]. This is in accordance with autopsy findings which suggests that an increase in myocardial mass during both LV and RV hypertrophy is associated with a proportional increase in EAT mass [8]. Increased LV mass and LV hypertrophy are independent risk factors for cardiovascular and all-cause mortality, so it can be supposed that the increment of EAT causes additional mass on both ventricles, which can enhance the cardiac work demands and lead to LV hypertrophy. Recently, innovative methods such as speckle tracking echocardiography (STE) or cardiovascular magnetic resonance (CMR) have allowed to study cardiac mechanics like strain, torsion, and synchrony of contraction, and thus highlight association of EAT volume and subtle abnormalities in cardiac structure and contractile function. In a study using STE, EAT was associated with longitudinal STE LV-dys-synchrony, longitudinal strain, circumferential LV-dys-synchrony, and LV twist [92] and in another study using CMR, in obese children, LV mass index, thickness, ejection fraction and peak longitudinal and circumferential strains were all correlated with EAT [93].

#### 2.4.4. EAT and COVID-19 

Given that obesity has been identified as an independent risk factor for complications and mortality in coronavirus disease 2019 (COVID-19), great interest has been shown in the involvement of EAT in this disease. EAT would appear to express higher levels of ACE2 than subcutaneous adipose tissue, which could make it a preferred viral reservoir [94]. Several studies have shown that EAT is a major driver of COVID-19 severity [94]. Using computed tomography (CT) scans and semi-automatic software, Cosson et al. demonstrated that volume was associated with the severity of COVID-19 and with transfer to intensive care unit (ICU) or death [95]. Iacobellis et al. showed that the density of EAT, reflecting inflammatory status, increased with rising COVID-19 severity [96]. Furthermore, the authors observed that EAT density was significantly reduced after treatment by dexamethasone, suggesting that EAT could be targeted by anti-inflammatory treatment [97]. Moreover, numerous studies have highlighted the possible implication of EAT in myocardial inflammation through its anatomical and functional relationship with the myocardium [94,98,99]. The EAT inflammatory secretome, such as interleukin-6 (IL-6), cytokine found in excess in severe COVID-19 patients, may be a key element in cardiac complications [98]. 

### 2.5. Effect of Exercise, Weight Loss, Pharmacological Intervention on EAT

Multiple studies have revealed that exercise, bariatric surgery and pharmacological intervention can reduce EAT volume [100,101,102,103]. Our team demonstrated a significant reduction of EAT amount after bariatric surgery [100]. We should underline that the EAT decrease was less important than the decrease in VAT. We also evaluated the EAT volume by MRI after pharmacological intervention. In a randomized type 2 diabetes (T2D) patient study, comparing exenatide (a glucagon-like peptide-1 agonist) versus reference treatment of T2D, according to French guidelines, we found a significant reduction of EAT volume after 26 weeks of treatment in the exenatide group in comparison to reference treatment [101]. Other treatments like statins [104] or SGLT-2 inhibitors [105,106,107] showed also a significant depletion of EAT. Some studies have found a benefit of physical exercise on EAT reduction in overweight subjects [102,103]. In a meta-analysis, it was demonstrated that supervised endurance training in particular decrease EAT amount with no involvement of total duration of the training [102]. However, Jonker et al. [108], found no difference after 6 months of preparation for a 12-day trekking expedition, which raises questions about the exercise effect. In total, all of these interventional studies focus on the changes of EAT volume without assessing changes in EAT characteristics such as its inflammation profile and its browning. What is still unknown is whether this reduction of EAT is associated with a modification in its composition or not. The diminution of EAT thickness might be related to the reduction of its inflammatory status, which has been suggested by a recent study performing a secretome analysis on EAT biopsies of patients under statins, but the results are too scarce and more investigations are needed [109]. Recently, using perivascular Fat Attenuation Index (FAI), an AI intelligence tool in CT scan that captures attenuation gradients of EAT surrounding coronary arteries (perivascular adipose tissue PVAT), Antoniades et al. analyzed the impact of pharmacological interventions on PVAT inflammation [110]. The treatment of psoriasis with anti-inflammatory antibodies (anti-TNFa, anti-IL17, anti-IL12/23) showed a reduction in FAIpvat values, which means a reduction in EAT inflammation [111]. Hence, this tool could be used to assess the inflammatory status of the diminished EAT. However, if this index reflects also the change in browning is not known and needs complementary studies.

## 3. The Browning of Adipose Tissue

Until a few years ago, white and brown adipocyte tissues were the main adipose tissues found and studied in mammals. One of the principal functions of the WAT is to store a large number of nutrients, particularly lipids in the form of triglycerides that can be released as fatty acids when food becomes scarce [112]. This is probably why these cells contain a large vacuole capable of storing lipids. The BAT which is a natural defense system against hypothermia in mammals [113,114,115,116,117], is composed of smaller multilocular lipid droplets and numerous mitochondria. Indeed, WAT and BAT are dynamic tissues capable of a form of adaptation. In fact, they can respond to different forms of stress such as cold exposure for the BAT, or even starvation or overfeeding for the WAT [118,119,120]. The BAT is mainly defined as a heat producer [120]. The particularity of the BAT is that it can dissipate the energy in the form of heat, this process is called non-shivering thermogenesis and is the mean difference between WAT and BAT [120]. This thermogenic function comes from UCP-1 [121]. This protein inserted in the inner membrane of the mitochondria can be activated by cold exposure or food intake and acts as a proton channel that dissipates the electrochemical gradient produced by the oxidative phosphorylation without allowing the synthesis of ATP, the energy is then released as heat [122,123]. Recent studies using positron emission tomography (PET) reported the presence of metabolically active BAT within the neck and upper chest regions of human adults [124,125]. Thus, BAT has been shown to be an important regulator of energy expenditure, and a potential therapeutic target in obesity. But there are other adaptive changes that can be found in WAT and which are related to the thermogenic function of BAT. It is indeed possible in certain conditions to observe the appearance of ‘brown-like’ adipocytes in WAT [4,5,6].

A common definition of browning is the induction of a thermogenic function by WAT. These cells are then named beige or brite (brown-in-white) adipocytes and were first found in cold-acclimated mice [126], suggesting that WAT can acquire thermogenic properties. Beige adipocytes are characterized by multiple lipid droplets containing triglycerides and numerous mitochondria. These changes can occur when the cells are activated by thermogenic stimuli such as cold exposure [127,128] or stimulated by browning factors such as β3-adrenergic agonists [129,130,131,132,133,134], or glucagon like peptide 1 (GLP-1) agonists [135,136,137,138]. 

Beige adipocytes are mostly characterized by their expression of UCP-1 just like brown adipocytes but display distinct molecular signature from brown adipocytes in mouse and humans [127]. It has been evidenced that BAT comes from Myf5+ lineage precursors while beige adipocytes precursors are Myf5− and PDGFRα+ [127,139]. Moreover, to prove that beige and brown adipocytes are quite distinct, Wu et al. have studied beige cells from murine white fat depots [127]. They observed a gene expression profile distinct from white or brown fat in these beige adipocytes. But although a beige cell lineage exists, evidence of trans-differentiation from white to beige adipocytes in vivo has also been evidenced [140]. 

### 3.1. White, Brown, and Beige Adipocytes Markers

**Figure 2:**
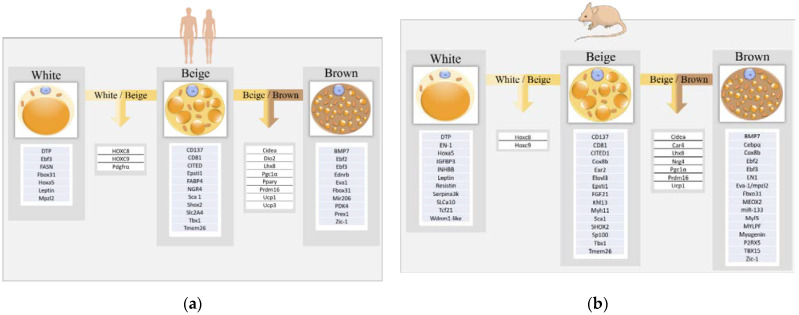
White, Beige and Brown adipocyte markers in Human and Mouse. We summed up here genes reported in the literature as markers of white (WAT), beige and brown adipose tissues (BAT) and shared by beige and WAT or beige and BAT in humans (**a**) and mice (**b**).

In order to better understand the mechanisms of browning, it is essential to be able to distinguish the different subtypes of adipocytes. For this purpose, there are many markers for white, brown and beige adipocytes in mice and humans. In this review, we tried to sum up the majority of these markers in Figure 2. 

#### 3.1.1. White Adipocyte Markers

To begin, a common marker used in human and mice for white adipose tissue analyses is Leptin [141,142]. In mice subcutaneous WAT, it has been shown that white adipocytes expressed Asc-1 (also called solute carrier family 7 member 10 (SLC7a10)) specifically [143,144], but also the serine protease inhibitor A3K (Serpina3K), *Wndnm1-like* [144,145] and *Tcf21* [146]. In humans, others specific WAT genetic markers are known, among them are *Ebf3*, *Fbox31* and *Mpzl2* [147], and *FASN* [148]. Although WAT and BAT do not currently share markers, white and beige adipocytes from mice and humans have in common *Pdfrα* [149,150] and the homeobox *(Hox)C8* and *Hoxc9* genes normally used to identify white adipocytes [144,146,150].

#### 3.1.2. Brown Adipocyte Markers

Since it was known that both beige and brown adipocytes have common characteristics, it was necessary to distinguish them and discover which markers they had in common and which were really specific to brown adipocytes. For this purpose, De Jong et al. evaluated the expression of several markers in interscapular (BAT), inguinal (beige), and epididymal (WAT) mice adipose tissues and found that only Zic-1 mRNA was detectable in BAT [151]. Others studies have also come to this conclusion with BAT gene expression profile studies in mice but also in humans [146,147,152,153]. In human BAT and mice interscapular region, studies identified Eva1 (also known as Mpzl2) as another specific BAT marker, with significantly higher expression in this tissue compared to beige and white adipose tissues [127,154]. Ussar et al. demonstrated that the expression of *P2RX5* is up-regulated in brown preadipocytes and adipocytes and that its expression is further increased during cell differentiation [143]. Ancient markers that were commonly used to identify BAT, have been shown to be expressed by beige cells as well. It is now well-known that UCP-1 can also be expressed by beige adipocytes, but other markers shared by both cell types such as *CIDEA* [144,145], *Lhx8* [144], *PGC1α* [155,156], *PRDM16* [150,154,157] have been evidenced and many others as listed in Figure 2.

#### 3.1.3. Beige Adipocyte Markers

In order to evaluate markers of mice beige adipocytes, Garcia et al. studied transcript expression of several thermoregulatory genes and proposed specific beige markers such as *Cox8b* [145] or the fibroblast growth factor 21 (*FGF21*) [145,158]. In their transcriptomic study, De Jond et al. also analyzed potential beige markers and identified *CD137*, *Epsti1*, *Tbx1* and *Tmem26* as being quite specific of beige adipose tissue [151]. Although reservations have been made on *Tbx1* and *Tmem26* because a comparison of their expression in brown and white adipocyte cell cultures revealed no qualitative difference [144]. In addition, CD137 and Tmem26 are known to be cell surface markers, notably for beige precursors [159], which constitute a major advantage in the isolation of these cells from WAT. Recently, Comas et al. discovered a novel marker of beige adipocytes called neuregulin 4 (NRG4) in human adipose tissue [148]. This study also showed a significant relationship between NRG4 and TMEM26 gene expression in both VAT and SAT. Other markers reported to be specific to beige adipose tissues have been reported, in both humans and mice, such as *CITED1*, *Ear2*, *Elov3*, *Sca1, Dio2* (Figure 2).

### 3.2. Browning Factors

#### 3.2.1. Thermogenic Stimuli

Many stimuli contribute to the apparition of beige adipocytes in WAT. Using positron emission tomography (PET) scan studies showed that cold exposure is one of the main sources of BAT activation and browning induction [124,160,161,162,163]. In response to a cold stress, sympathetic nerve terminals will release catecholamines, such as norepinephrine (NE), that will be addressed to their β-adrenergic receptors (β-AR). The involvement of the β3-AR in the browning has been demonstrated many times, and it has been found that chronic treatment with β3-adrenergic agonists induces the browning of WAT [130,164,165,166,167]. More recently, a study investigated the physiological signals involved in cold-induced browning in mice showed that white adipocytes can receive adrenergic signals via β3-AR and produce FGF21 which stimulates eosinophils and M2 macrophages. The stimulation of these type 2 immune cells leads to the browning of SAT but not BAT or epidydimal adipose tissue [158]. Moreover, beige adipocytes seem to be able to remember if they have already been exposed to cold, a thermogenic capacity that they can thus re-employ more quickly [168].

#### 3.2.2. Proteins Stimuli

Another stimulus inducing the presence of beige adipocytes is the inactivation of the AMP-activated protein kinase (AMPK) in the ventromedial hypothalamus. This protein activated when cellular energy is depleted, can promote ATP-producing processes [169,170]. Its inactivation results in an increase sympathetic output to WAT thus inducing BAT activation and WAT browning [171]. The AMPK inactivation that leads to browning can be induced by thyroid hormones [172,173] or glucagon like peptide 1 receptor (GLP-1R) agonists [137]. 

#### 3.2.3. Lipids Stimuli

Recently, lipids have also been identified as regulators of thermogenic fat activation notably via a specific crosstalk with sympathetic neurons [174]. Indeed, growing evidence suggests that de novo lipid synthesis through the fatty acid synthase (FASN) mediates the expansion of beige adipocytes within inguinal white adipose tissue [175]. Another lipogenic pathway related to lipids was found to modulate PPARγ activation of brown-like adipocytes in mice SAT, the peroxisomal reductase activating PPARγ (PexRAP) [176]. Other evidences of lipid involvement exist, for example the polyunsaturated fatty acid, eicosapentaenoic acid (EPA), has been shown to promote BAT thermogenic capacity by increasing the UCP1 content amplifying catecholamines such as NE-stimulated oxygen consumption [177,178]. In mice, EPA has also been shown to increase UCP-1 gene expression, enhancing the thermogenic response of BAT and inguinal WAT to β3-adrenergic stimulation [178,179]. Park et al. further demonstrated that peroxisome-derived lipids, including plasmalogens, are able to regulate adipose thermogenesis by mediating cold-induced mitochondrial fission in brown and beige adipocytes [180]. 

#### 3.2.4. Natriuretic Peptides 

Atrial natriuretic peptide (ANP) and brain natriuretic peptide (BNP) are endocrine hormones released from the heart in response to cardiac wall stress and other local factors [181,182,183,184]. Several studies have showed that cardiac natriuretic peptides are browning inducers. In both humans and mice adipocytes, it has been demonstrated that natriuretic peptides activate the mammalian target of rapamycin complex 1 (mTORC1) signaling [185]. This last complex has been shown to be necessary for cold-induced browning [186,187]. In addition, ANP treatments significantly increased UCP-1 expression, ameliorated high fat diet-induced insulin resistance in mice by inducing adipose tissue browning [188]. Bordicchia et al. also demonstrated that in human adipocytes, ANP and BNP activated PPARγ coactivator-1α (PGC-1α) and UCP-1 expression, induced mitochondriogenesis, and increased uncoupled and total respiration in a p38 MAPK-dependent manner. Further infusion of BNP into mice robustly increased Ucp1 and Pgc-1α expression in WAT and BAT, with corresponding elevation of respiration and energy expenditure confirming that natriuretic peptides can promote browning of WAT to increase energy expenditure, defining the heart as a central regulator of adipose tissue biology [189].

#### 3.2.5. Extracellular Vesicles (EVs) and miRNA

Several adipose tissue cell subtypes including adipocytes, adipose tissue-derived stem cells (ADSCs), endothelial cells and macrophages have been reported to secrete extracellular vesicles (EVs) [190]. Adipose-derived EVs include exosomes, microvesicles and apoptotic bodies [191]. As mentioned earlier, EVs have emerged as a new way of inter-organ and intercellular communication in the EAT/cardiovascular system crosstalk. They are now also known to be involved in WAT browning. In 2018, a unidirectional transfer of exosomes from adipose tissue-derived stem cells (ADSCs) to macrophages has been observed [192]. Moreover, these exosomes have been shown to induce the polarization of macrophages into the anti-inflammatory M2 subtype. The authors also demonstrated that these ADSCs-derived exosomes promote inguinal and epididymal WAT-browning in mice. In 2020, these findings have been transposed to human ADSCs-derived exosomes [193]. Secretion of exosomes during stem cell differentiation into white or beige adipocytes can promote cell reprogramming implying that beige adipocytes-derived exosomes can stimulate the development of other beige cells in WAT. Although these findings remain interesting, it is now necessary to discover which component(s) of ADSCs-derived exosomes is/are responsible for browning. Indeed, in addition to containing nanovesicles, proteins, bioactive lipids or non-coding RNAs, they also enclose microRNAs (miRs) [190,194]. Some of them have been shown to have a role in WAT browning. miR-196a has been shown to induce WAT-browning during cold exposure and β-adrenergic stimulation [195]. miR-155 increased brown adipose tissue function and leaded to a brown adipocyte-like phenotype in white adipocytes [196]. Therefore, further studies are required to identify the precise involvement of EVs and their still poorly understood content in the biogenesis of beige adipocytes and in the white-to-beige differentiation.

#### 3.2.6. Muscle

We have to highlight the crosstalk between adipose tissue and muscle in promoting the browning of adipose tissue. Indeed, skeletal muscle and adipose tissue secrete METRNL (Meteorin-like), a PGC-1α-dependent myokine, in response to physical exercise and cold exposure [197]. Rao et al. showed that METRNL can enhance browning without acting directly on adipocytes [198]. In fact, METRNL was found to induce the recruitment of eosinophils in WAT leading to an increased expression of IL4 and IL13, which results in stimulation of M2 macrophages and activation of thermogenic genes. Irisin, a cleavage product of Fndc5 gene (fibronectin type III domain containing 5), is another exercise-stimulated PGC-1α-dependent myokine implicated in the browning of WAT [199] but its secretion by the skeletal muscle, its regulation and function are a source of discrepancy in the literature [200].

#### 3.2.7. Immune Cells

In addition to the previous browning factors, many immune actors have been found to be involved in the appearance of beige adipocytes in WAT. Among these actors, type 2 immune cells and their related type 2 cytokines could play a major role in the biogenesis of beige adipocytes. Lee et al. reported in their work on thermoneutral mice, that activation of type 2 innate lymphoid cells (ILC2s) by interleukin (IL)-33 is sufficient to promote the growth of adipocyte precursors committed to the beige fat lineage [201]. In fact, they reported that a thermogenic circuit exists activating ILC2s which in response secrete IL-13. The authors then showed that this ILC2-derived cytokine in cooperation with the eosinophil-derived IL-4 will directly promote the expansion and commitment of beige adipocyte progenitors in the SAT through the IL-4 receptor α (IL-4Rα) signaling. In 2014, ILC2 were also identified in human WAT by Brestoff et al. [57] and shown to be decreased in obesity. Moreover, IL-33 was found to be critical for the maintenance of ILC2s in WAT and for the induction of browning. To go further into the contribution of ILC2 in browning, they focused on obesity-associated genes they expressed. They particularly observed that ILC2 could product methionine-enkephalin peptide (MetEnk), the latter having the ability to increase beige adipocytes in WAT. Moreover, one of the receptors for MetEnk, δ1 opioid receptor (Oprd1), was highly expressed in inguinal WAT suggesting that there may be tissue-specific effects of MetEnk in WAT. A recent study combining neural and immune systems also established that the sympathetic nervous system can activate PDGFRα positive mesenchymal cells, present in the stromal vascular fraction, through the β2-adrenergic receptor. In response to sympathetic signals, these cells secreted the glial-derived neurotrophic factor (GNDF) that was able to modulate the activity of ILC2s via the tyrosine kinase receptor RET. In this study, Cardoso and colleagues further demonstrated that RET was essential in ILC2s activities especially in the secretion of IL-5, IL-13 and MetEnk that will participate to the shaping of energy expenditure, host metabolism and obesity. 

Recent literature also suggest that other innate lymphoid cells could be implicated in the browning. Among them are eosinophils. First, a study demonstrated the implication of eosinophils in the activation of alternative activated macrophages (AMMs) by expressing IL-4 in perigonadal WAT [202]. Moreover, they showed that these eosinophils were the major IL-4-expressing cells in mice perigonadal WAT. They further demonstrated that eosinophils migrate from blood into WAT by an integrin-dependent process and reconstitute AAMs through an IL-4/IL-13-dependent process. Then, another study showed that eosinophils is a part of the efferent branch from the thermogenic circuit that regulates cold-induced browning of SAT [203]. In 2011, it has been shown that after their activation by IL-4, AMMs are able to modulate adaptive thermogenesis [204]. The Huang et al. study cited above showed that cold exposure can induce an autocrine signaling of FGF21 in mature white adipocytes [158]. This leads to the expression of the chemokine CCL11 activating eosinophils which in turn secrete IL-4 that can either activate M2 macrophages or induce the browning of PDGFRα positive adipocyte precursors. Moreover, the formation of beige adipocytes can also result from the existence of a genetic or environmental background or from fat depot specific differences [205]. 

Finally, in 2018 a study highlighted the role of γδ T cells in adipose tissue thermogenesis. The authors demonstrated that IL-17A-producing γδ T cells interact with adipose stromal cells and consequently regulate IL-33 abundance, which affects Treg cell accumulation and thermoregulation [60]. They also showed that γδ T cells and IL-17A deficiencies significantly affect the ability of mice to survive after a cold challenge and strongly induce UCP-1-dependent thermogenic responses. This advance in the dynamic crosstalk between innate lymphoid cells and adipose tissue dictates the local immune composition responsible for adipose tissue wiggling and thermogenesis.

## 4. Browning EAT: Friend or Foe?

### 4.1. Evidences of EAT Beige Phenotype

Before the appearance of the term of beige adipocytes in the EAT, researchers had described EAT as functioning like a brown adipose tissue [7,14]. Sacks et al., in a transcriptional analysis of BAT markers in human EAT, concluded that, since UCP-1, PRDM16, and PGC-1α were expressed more strongly in this tissue than in other fat depots, EAT could function as brown fat. This could serve to defend the myocardium and coronary vessels against hypothermia and protect the heart from ischemia or hypoxia [14]. In 2013, another study of the EAT gene expression profile revealed the presence of the beige adipocyte-specific marker CD137, and histological analysis showed small unilocular adipocytes in the tissue [3]. This same study showed significantly increased levels of UCP-1 in mitochondria from EAT compared to paracardial, abdominal, and sternal SAT. Moreover, the authors found that those EAT UCP-1 concentrations were comparable to those found in pericardial BAT sampled from newborn sheep in which the protein is maximally expressed and activated. The same year, in a cohort of CAD patients, elevated mRNA expression of UCP-1 in EAT was shown and a correlation with circulating lipid levels was made [206]. Since growing evidence suggests that lipids play a role in the browning phenomenon [207], we performed an untargeted lipidomic study on EAT and SAT from CAD and non-CAD patients and compared them with paired plasma lipidomic analysis of isolated VLDL (very low-density lipoprotein). This work showed for the first time that EAT and SAT had independent lipidomic profile. Secondly, we found that six plasmalogen species were significantly enriched in EAT compared with SAT. These specific plasmalogens increase could reflect a thermogenic activity of the EAT compared to SAT [207]. Indeed, Park et al. suggested that manipulation of plasmalogen production by dietary or pharmacological approaches could enhance the thermogenic status of beige adipocytes [180]. Adipose-specific KO of the peroxisomal biogenesis factor Pex16 (Pex16-AKO) in mice impaired cold tolerance, decreased energy expenditure, and increased diet-induced obesity. Pex16 deficiency blocked cold-induced mitochondrial fission, decreased mitochondrial copy number, and caused mitochondrial dysfunction. This highlighted that peroxisome-derived lipids regulate adipose thermogenesis by mediating cold-induced mitochondrial fission. Therefore, function of plasmalogens in EAT deserves more research and the possible plasmalogens-induced browning of the EAT further investigations. Other dietary treatments have been suggested to be implicated in EAT browning such as aged garlic extract. Ahmadi et al. showed that aged garlic extract was associated with increased brown EAT, and prevented the progression of CAC score [208].

Using a pangenomic approach, our research group has added further evidence of the human EAT beige profile [2]. Indeed, an EPICAR study aimed at determining the specific transcriptomic profile of EAT. We showed that the peri-ventricular EAT could be very sensitive to browning. In particular, we showed that EAT expressed some BAT specific genes such as UCP-1 actin alpha 1 (ACTC1) or PPAR gamma co-activator 1 alpha (PPARGC1A). Moreover, WAT specific gene *HOXC9* was significantly downregulated in EAT compared with SAT. All this suggests that EAT exhibits a beige phenotype. More recently, another study using next-generation deep sequencing compared gene signatures from EAT, SAT and mediastinal adipose tissue (mAT) [15]. In this work, the authors identified lipid metabolism-related pathways associated with thermogenesis in EAT but not in mAT or SAT. These include fatty acid activation, mitochondrial L-carnitine shuttle pathway, fatty acid β-oxidation I and γ-linolenate biosynthesis. Association of genes involved in fatty acid oxidation and in white-to-brown fat differentiation with EAT glucagon like peptide 1 receptor (GLP-1R), has been depicted [209]. EAT expresses GLP-1R at both gene and protein levels and it has been shown that GLP-1 analogs (GLP-1A) has important cardiovascular beneficial effects that go beyond their antidiabetic actions, with a substantial reduction of EAT in diabetic and obese patients in patients treated with GLP-1A [101,210]. Dozio et al. who demonstrated the association between fatty acid oxidation genes and EAT GLP-1R showed that GLP-1A, by targeting EAT GLP-1R, may reduce local adipogenesis, improve fat utilization and induce brown fat differentiation [209]. Therefore, it is tempting to speculate on an intriguing strategy targeting GLP1-R to reverse metabolic derangement of EAT and future studies are warranted in this direction. 

### 4.2. Proposed Browning Factors within the EAT

**Figure 3:**
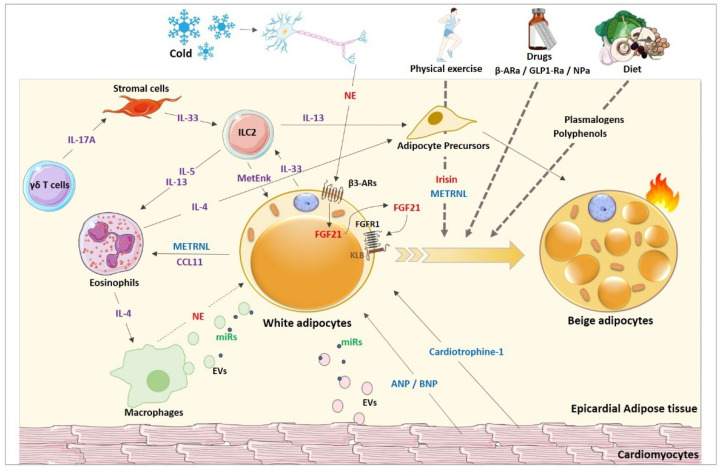
Potential factors involved in the EAT browning. Putative mechanisms based on work realized in adipose tissue in general. Environmental factors such as cold exposure, physical exercise, pharmacological treatments and dietary are implicated in browning of white adipocytes but the mechanisms underlying are not yet well established (dotted arrows). Inter-cellular communication factors involving cytokines/chemokines (purple), myokines (blue), hormones (red) and miRs (green) are known to be involved in WAT browning. EVs: extracellular vesicles; β-Ara: β-adrenergic receptor agonists; GLP1-Ra: GLP1- receptor agonists; NPa natriuretic peptide agonists; METRNL: Meteorin Like; MetEnk: methionine enkephalin, NE: norepinephrine, FGF21: fibroblast growth factor 21; FGFR1: FGF receptor 1; KLB: coreceptor Beta-klotho.

Interestingly, Chechi et al. also reported that EAT displays an overrepresentation of immune-related pathways that brings it closer to mAT than to SAT [15]. Moreover, they found that EAT was enriched in T-cell related pathways such as ICOS-ICOSL signaling, T helper cell differentiation, or Th2 pathway. This last signaling pathway has been since few years widely studied in the implication of browning. Therefore, it is possible to imagine that these immune related pathways can contribute to the epicardial fat browning (Figure 3). Unfortunately, not many studies have yet demonstrated the involvement of these cells in the EAT beige status. Recently, researchers have established that a TNF superfamily member, the death receptor 3 (DR3), was expressed in murine VAT and human peripheral blood-ILC2s and inducible by IL-33 [211]. They also revealed that mice treated with DR3 agonist exhibited significantly enhanced expression of Ucp1, Cidea, Prdm16, Pgc1a and Dio2 at the transcriptomic level. Whether these factors could have a direct paracrine effect on EAT remains to be determined.

One evidence of the direct type 2 immunity involvement in the EAT is the Sacks et al. study from 2011 [28], complemented by the study of Distel et al. from 2012 [212]. The first study showed increased expression of anti-inflammatory interleukin-1 receptor antagonist (IL-1Ra) and IL-10, in EAT from metabolic or T2D subjects suggesting a potentially beneficial role for these adipokines in a proinflammatory milieu contiguous with CAD. Treatment with pioglitazone in T2D patients with CAD was associated with a reduction of proinflammatory and anti-inflammatory genes in EAT and a selective increase in PPARγ in SAT. The second study investigated the short-term effect of rosiglitazone on the expression of the genes and proteins (by RT-PCR and Western blot) involved in fatty acid metabolism in EAT of the obese fatty Zucker rat and compared the levels of expression with those in retroperitoneal adipose tissue. Interestingly, the expression of the genes encoding proteins involved in mitochondrial processing and density PPARγ coactivator 1 alpha (PGC-1α), NADH dehydrogenase 1 and cytochrome oxidase (COX4) were increased by rosiglitazone only in EAT, with a resulting significant up-regulation of PGC1-α and COX4 protein. This was accompanied by a rise in the expression of PRDM-16 and UCP-1, revealing that this PPAR-γ agonist could induce a rapid browning of the EAT that probably contributes to high lipid turnover in this tissue.

Finally, it has recently been demonstrated that EAT adipocytes can release EVs that can penetrate cardiac cells by endocytosis [25] (Figure 3). Very recent data have evidenced functional mitochondrial transfer from energetically stressed adipocytes to the heart that could limit cardiac ischemia/reperfusion injury in mice and prevent lipotoxicity [213,214]. Potential factors implicated in the EAT browning are summarized in Figure 3.

It may hence be supposed that re-browning of EAT in obese and/or CAD patients, using a diversity of dietary, environmental and pharmacological approaches, may improve the hypoxic, inflammatory microenvironment disturbing the vasculature and contributing to coronary atherosclerosis [215]. However, the factors specifically involved in EAT browning and regulating this phenomenon remain to be clearly identified, and other experimental studies are needed to better understand which pathway could be targeted to improve the phenotype of EAT and hence reduce the cardiovascular outcomes associated with its imbalanced inflammatory phenotype.

### 4.3. EAT Whitening as Foe

There is a large body of evidence supporting the effects of browning activation in improving obesity and its cardiac complications. The opposite phenomenon brown-to-white trans-differentiation, also referred as ‘whitening’, has been far less explored. Under physiological (as aging) and pathological circumstances, epicardial adipocytes may lose those cardioprotective functions and turn into pro-inflammatory cells. As people get older, the proportion of brown adipocytes decreases in favor of more unilocular white adipocytes and so EAT brown fat-like activity and function could decrease with first the loss of its thermogenic properties [216]. This is the brown-to-white trans-differentiation. Hence, aging can influence the function of EAT partly because of transition from brown to beige fat of EAT in adult life. But if in one hand, this phenomenon can help thermogenic homeostasis, in the other hand in some cases it can have less beneficial effects. In pathophysiological cases where browning can have beneficial consequences, this brown-to-white trans-differentiation could reverse these positive influences. For instance, in EAT this brown-to-white trans-differentiation has been associated with an increased reactive oxygen species (ROS) production in CAD patients [74].

EAT browning characteristics can also be impaired in advanced chronic diseases. Dozio et al. showed that EAT of patients with CAD was associated with decreased expression levels of thermogenic genes and upregulation of white adipogenesis [74]. Indeed, they demonstrated that the BAT-specific genes UCP-1, PGC-1α, PRDM16 and bone morphogenetic protein 7 (BMP7) were statistically significant lower in CAD patients and WAT-specific gene insulin-like growth factor binding protein 3 (IGFBP3) and HOXC9 were significantly higher compared to non-CAD patients, which suggests a change in the composition of the EAT in CAD patients. 

Other possible cardioprotective pathway genes, involving PGC-1α, involved in adipocyte browning and thermogenic activation, have been suggested. Epicardial fat heme oxygenase-1 (HO-1) PGC-1α may modulate inflammation, mitochondrial activity and left ventricle function. A decrease of HO-1, PGC-1α and PRDM16 in EAT compared to VAT was linked to significant cardiac remodeling and was observed in cardiomyopathies [217]. It has been suggested a more WAT-like genotype at EAT level in CAD patients [74]. EAT seems to produce less ROS than SAT, but when comparing CAD patients with non-CAD patients, only non-CAD patients have a significantly higher ROS level in SAT than in EAT. In addition, there is a significant higher ROS level in EAT of CAD patients compared to non-CAD patients. All these results support the hypothesis that CAD may be associated with brown-to-white trans-differentiation process. 

### 4.4. Perspectives: Non-Invasive Imaging Techniques

One of the major limitations in studying the EAT is that rodents have no EAT. In Humans, the EAT study from fresh tissue is also limited due to the difficulty of obtaining samples because this needs thoracotomy. Thus, imaging techniques have become valuable tools in the understanding of EAT pathophysiology either in terms of volume quantification or functional characterization, two parameters that might improve the individual cardiovascular risk stratification. 

Artificial intelligence (AI) tools using deep learning approach, applied to CT scans, allow to speed up the first stages of image pre-processing and allow the improvement of quantification and volume segmentation of EAT [218]. The length of execution does not exceed 26 seconds and there is a high correlation between automated and manual measures [219]. 

Vascular inflammation is a key component of the atherosclerotic process, and has been shown to induce molecular, transcriptional and structural changes to perivascular fat. Regarding the functional aspect of EAT, detection of pericoronary EAT inflammation, is now possible with non-invasive imaging tools, such as CT scan [110,218]. The perivascular Fat Attenuation Index (FAI), an imaging tool that measures weighted 3D attenuation gradients of AT in the perivascular space, involves the use of AI-enhanced algorithms that provide accurate and reproducible weighted measures of attenuation in 1mm 3D layers of EAT around the human arterial wall. This FAI has the potential to yield informative results on local coronary inflammation and plaque vulnerability. [110,220]. Detecting these changes of composition could have great clinical implications. In the CRISP-CT study [221,222], patients with the highest FAI values of coronary vessels had a significantly higher risk of all-cause mortality and cardiac mortality [221].

Unfortunately, to date there are still no specific tools to evaluate the browning of the EAT. In animals, new techniques have emerged to quantify the browning by using, for instance, Magnetic Resonance Imaging (MRI) [223] or Positron Emission Tomography (PET) [224], but all these experiments were realized on mice. Regarding humans, the BAT can be studied by several imaging techniques. The [18F]fluorodeoxyglucose ([18F]FDG)-PET /CT imaging with a cold exposition during 2 hours is the best established method for visualizing activated BAT in humans [225]. However, this technique exposes subjects to ionizing radiation and requires a lot of equipment with a high cost. Furthermore, the BAT detection cannot be done without stimulations (cold temperature or drug induction) and FDG uptake by the myocardium prevents from EAT browning detection [226,227]. Moreover, it should be noted that glucose tracer does not provide information on total BAT oxidative metabolism, but the addition of fatty acid tracers and labeled oxygen tracers could provide missing information [228]. Compared to PET and SPECT, MR imaging is a more attractive modality to investigate BAT. It does not expose to ionizing radiation and both volume and function can be studied. Chemical shift MRI such as fat fraction mapping and T2*-weighted mapping were able to measure BAT volume while Blood Oxygen Level Dependent (BOLD) MRI, hyperpolarized Xenon MRI, and contrast-enhanced MRI have been employed to assess BAT function [226,227]. Moreover, new imaging techniques, such as Near-Infrared Spectroscopy (NIRS) techniques and Infrared Thermography (IRT) are being developed for BAT imaging. These two techniques are relatively inexpensive and no cold exposure is required with NIRS techniques, though both of them, and especially IRT, cannot assess total BAT volume [226,227]. One important issue is that complete determination of the thermogenic potential of human BAT requires not only assessment of BAT following acute stimulation, but also BAT in its basal state. A single imaging method could have limitations to accurately report BAT mass and BAT activation at the same time, and a combination of different methods or modalities could be the trend for monitoring both the BAT mass and metabolic state change in future research. Despite limitations, all these techniques are promising ways for a future non-invasive evaluation of human EAT browning.

## 5. Conclusions

EAT is a unique depot in direct contact with coronary arteries and myocardium that can probably drive heart diseases. The beige adipocytes factors and immune systems cross-talks are fascinating topics to explore in order to better understand the beiging mechanism in EAT and its impact on cardiac diseases. Furthermore, the anatomic proximity of the cardiac muscle which can secrete browning cardiokines makes this cross-talk particularly appealing. New non-invasive techniques are needed to better follow EAT browning in humans after nutritional, or therapeutical interventions.

## Figures and Tables

**Table 1:** Summary of the presence or absence of immune cell subtypes infiltration within adipose tissues in the context of obesity and related complications. Arrows represent an increase (up arrows) or decrease (down arrows) of cells in EAT or VAT compared to SAT referenced in mice (red) and human (blue). N/A: not applicable; ND: not detectable.

Immune Cells			EAT		VAT	
			EAT	References	VAT	References
Innate immune cells	Macrophages	M1	↑	Hirata et al., 2011; Vianello et al., 2016; Gurses et al., 2017	↑	Wisnewsky et al., 2009; Aron-Wisnewsky et al., 2009; Morris et al., 2011
		M2	↓	Hirata et al., 2011; Vianello et al., 2016; Gurses et al., 2017	↓	Wisnewsky et al., 2009; Aron-Wisnewsky et al., 2009; Morris et al., 2011
	Eosinophils		N/A	-	↑	Wu D et al., 2011; Molofsky et al., 2013
	Mast cells		Presence	Laine et al. 1999; Mazurek et al., 2003	↑	Divoux et al., 2012; Shi and Shi 2012
	Neutrophils		N/A	-	↑	Carmon et al., 2008; Talukdar et al., 2012
	Natural killers		↓	Mráz et al., 2019	↑ ↑	O’Rourke et al., 2013; Trim et al., 2018; Lee et al., 2016; Wensveen et al., 2015
	Dendritic cells		↑	Mráz et al., 2019; Horcksman et al., 2017	↑ ↑	Berthola et al., 2012; Bapat et al., 2015
	Innate lymphoid Cells (ILCs)	ILC1	N/A	-	↑	Everaere et al., 2017
		ILC2	N/A	-	↓	Everaere et al., 2017
		ILC3	N/A	-	N/A	-
Adaptative immune cells	T Lymphocytes	LTreg	N/A	-	↑	Feuerer et al., 2009; Bapat et al., 2015
		CD4+	ND	Hirata et al., 2011	↑/↓	Nishimura et al., 2009; Lee et al., 2016
		CD8+	↑	Hirata et al., 2011	↑ ↑	Duffaut et al., 2009; Bapat et al., 2015
	B Lymphocytes		↑	Mráz et al., 2019	↑	Bapat et al., 2015
